# Protein Identification of Seminal Plasma in Bali Bull (*Bos javanicus*)

**DOI:** 10.3390/ani13030514

**Published:** 2023-02-01

**Authors:** Hikmayani Iskandar, Göran Andersson, Herry Sonjaya, Raden Iis Arifiantini, Syahruddin Said, Hasbi Hasbi, Tulus Maulana, Abdullah Baharun

**Affiliations:** 1 Agricultural Science Study Program, Graduate School Hasanuddin University, Makassar 90245, Indonesia; ihikmayani@gmail.com; 2 Department of Animal Breeding and Genetics, Swedish University of Agricultural Sciences, 75007 Uppsala, Sweden; goran.andersson@slu.se; 3 Department of Animal Production, Faculty of Animal Science, Hasanuddin University, Makassar 90245, Indonesia; hasbi_fapetunhas@yahoo.com; 4 Department of Veterinary Clinic, Reproduction and Pathology, Faculty of Veterinary Medicine, IPB University, Bogor 16680, Indonesia; arifiantini@apps.ipb.ac.id; 5 Animal Repronomics Research Group, Research Center for Applied Zoology, National Research and Innovation Agency, Bogor 16914, Indonesia; syahruddinsaid01@gmail.com (S.S.); tulus_maul@yahoo.com (T.M.); 6 Animal Science Program, Faculty of Agriculture, Djuanda University, Bogor 16720, Indonesia; abdullah.baharun@unida.ac.id

**Keywords:** Bali bull, seminal plasma, protein, fertility, biomarkers

## Abstract

**Simple Summary:**

In this study, in order to provide a better understanding of and new knowledge about seminal plasma protein in Bali bull (*Bos javanicus*), we analyzed plasma semen using LC-MS/MS. The results of the analysis were then analyzed using bioinformatics to explore the total protein, expression, and inferred function of these seminal plasma proteins of Bali bulls. We identified six proteins that have previously been associated with fertility. These studies identified seminal plasma proteins that will have potential as biomarkers of fertility in Bali bulls.

**Abstract:**

The purpose of this study was to identify seminal plasma proteins in Bali bull and their potential as biomarkers of fertility. Semen was collected from 10 bulls aged 5–10 years using an artificial vagina. Fresh semen was then centrifuged (3000× *g* for 30 min). The supernatant was put into straws and stored in liquid nitrogen. The semen plasma protein concentration was determined using the Bradford method, and the protein was characterized using 1D-SDS-PAGE. Coomassie Brilliant Blue (CBB) was used to color the gel, and the molecular weight of the protein was determined using PM2700. A total of 94 proteins were identified in the seminal plasma of Bali bulls analyzed using LC-MS/MS (liquid chromatography–mass spectrometry). Proteins spermadhesin 1 (SPADH1), C-type natriuretic peptide (NPPC), clusterin (CLU), apoliprotein A-II (APOA2), inositol-3-phosphate synthase 1 (ISYNA1), and sulfhydryl oxidase 1 (QSOX1) were identified as important for fertility in *Bos javanicus*. These proteins may prove to be important biomarkers of fertility in Bali bulls. These proteins are important for reproductive function, which includes spermatozoa motility, capacitation, and acrosome reactions. This study provides new information about the protein content in seminal plasma in Bali bulls. The LC-MS/MS-based proteome approach that we applied in this study obtained 94 proteins. The identification of these seminal plasma proteins of Bali bulls and their potential as fertility biomarkers may have an impact on the success of future artificial insemination (AI).

## 1. Introduction

Indonesia is a country consisting of 34 provinces, with a population of around 273 million people as of 2021. Nearly 4.5 million households in Indonesia raise livestock, and half of the farmers are small-scale cattle farmers [1]. Bali cattle are the most popular type to breed due to their easy maintenance, high fertility rate, resilience to tropical climatic conditions, sustainability on low-energy fodder, and ease of adaptation to the surrounding environment. *Bos sondaicus*, *Bos javanicus*, and *Bos*/*Bibos banteng* are domesticated cattle species of the wild banteng (*Bibos banteng*), and represent 27% of the total cattle population in Indonesia [2].

Bull fertility can be inferred from the results of artificial insemination (AI) through the proportion of cows that do not return to estrus after insemination and are considered pregnant. Using natural mating or AI to assess bull fertility is time-consuming and expensive because it cannot be carried out on many bulls at one time [3]. Bull fertility is very important to the success of AI. Therefore, the potential of the superior genetics of Bali cattle can be utilized. Seminal plasma has been known to be complex and consists of several different components (proteins, lipids, organic acids, minerals), each of which has a role in sperm function—many, but not all, have a well-defined function [4]. The different kinds of proteins from bulls with different fertility rates have not been fully defined or characterized. Semen is composed of about 5% testicular secretions containing spermatozoa and 95% seminal plasma, which includes secretions from accessory sex glands [5]. Seminal plasma has been shown to have a role in spermatozoa function and fertility by increasing the sperm’s viability and ability to move into the oviduct [6]. Furthermore, sperm quality is essential for bull fertility and seminal plasma proteins play an important role in sperm protection, capacitation, acrosome reaction, sperm–egg binding, fertilization, and early embryonic development [7,8,9]. The presence of specific proteins in seminal plasma may serve as potential biomarkers of fertility [5].

Several studies have reported the content and complexity of proteins in seminal plasma related to fertility [10,11,12]. Proteomics has been used to demonstrate differences in seminal plasma proteins derived from bulls of higher and lower fertility [13]. The proteomic approach is efficient for screening the total proteins in spermatozoa [14]. Proteomics has emerged as a technology in biomarker discovery [15,16]. Seminal plasma proteins are important for the maintenance of spermatozoa function [17]. Seminal plasma proteins have been characterized in sheep [18], *Bos taurus* cattle [19], pigs [20], buffalo [21], and horses [22]. Cell protection, motility, spermatozoa capacitation, acrosome reactions, fertilization, and embryonic development are some of the functions associated with seminal plasma proteins that have been previously reported [19]. 

The *Bos indicus* seminal plasma was analyzed, and eleven proteins were identified [23]. The relationship between seminal plasma protein and semen freezability from *Bos taurus indicus* and *Bos taurus taurus* was reported in [24]. The involvement and expressions of several proteins have been reported to affect spermatozoa motility, viability, acrosome integrity, capacitation, acrosomal reactions, and intracellular calcium concentrations [25]. Further studies of the seminal plasma proteome are therefore necessary to determine the proteins involved in male fertility. The assessment of protein characteristics based on proteomics was intended to detect important fertility-associated proteins in the seminal plasma of Bali bulls.

## 2. Materials and Methods

### 2.1. Ethical Approval

This study was approved by the Animal Ethics Commission of Hasanuddin University, Indonesia, license number: 302/UN4.6.4.5.31/PP36/2021.

### 2.2. Semen Samples

A total of 10 Bali bulls aged 5 to 10 years with spermatozoa motility >70%, based on secondary data from the Regional Artificial Insemination Center (RAIC) Puncak, Maros, South Sulawesi, Indonesia, were included in this study. The semen was collected using an artificial vagina. A total of 2 mL of semen was centrifuged at a speed of 3000 g for 30 min to separate the spermatozoa and seminal plasma. The supernatant was stored at −20 °C for further analysis.

Seminal plasma proteins were then extracted with a buffer containing 62.5 mM Tris-HCL (pH 6.8), 2% SDS, 1.0 mM phenyl methanesulfonyl fluoride, and 23 mM benzidine as a protease inhibitor. The seminal plasma was than vortexed for 10 min, followed by sonication [26].

### 2.3. SDS-PAGE and Staining Gel

Protein characterization was performed via 1D-SDS-PAGE based on molecular weight (MW). The separation of proteins was carried out using two 12% polyacrylamide gels containing sodium dodecyl sulfate (SDS). The mass of the protein (20 μg) was analyzed by SDS-PAGE with a PM2700 Excel-Band 3-color Broad Range (SMOBIO, Hsinchu, Taiwan). The MW ranged from 5 to 245 kDa.

Seminal plasma protein gels were stained with Coomassie Brilliant Blue [27,28]. Seminal protein concentration was determined using the Bradford method [29]. The Bradford protocol was performed according to the Coomassie Protein Kit Use Guide (Merck, Sigma-Aldrich, Darmstadt, Germany). Thermo Skanlt RE software Multiskan Go, Version 3.2 (Thermo Fisher Scientific, Waltham, MA, USA), was employed to examine the data.

The protein bands formed in the gel were measured and analyzed based on the protein range compared to the retention factor (Rf) walking interval. The results of the Rf analysis and the protein weight log of the band markers were transformed into linear regression equations. Protein separation was performed using 120 volts for 70 min, and the color photo gel was first inverted to allow for the easy identification of protein bands. The intensity differentiation of each protein band was determined by ratio analysis carried out using the ImageJ open source software [30].

### 2.4. Protein Quantification

A total of 25 g of aliquoted seminal plasma protein was placed in a microtube and dried in a vacuum. To each sample, 15 μL of lysis buffer containing 8 M urea, 0.02 M TEAB, and 0.5 M DTT (dithiothreitol) were added. This was followed by incubation at 55 °C and an agitation speed of 400 rpm (Eppendorf^®^ Thermomixer^®^ R, Sigma-Aldrich, Darmstadt, Germany) for 25 min. The lyophilization of the seminal plasma samples were suspended in 0.02 M TEAB, and the soluble protein was calculated using the Qubit^TM^ assay (Thermo Fisher Scientific, Waltham, MA, USA).

The alkylation process was then carried out by adding iodoacetamide (IAA) to reach a final concentration of 14 mM. The mixture was maintained at 21 °C and 400 rpm in the dark for 40 min. The digestion process was carried out by adding 5 mM DTT, 1 mM CaCl_2_, and 0.02 M TEAB for a final volume of 75 μL. The enzyme used in the digestion process was trypsin (Promega, Fitchburg, WI, USA), with an enzyme/substrate ratio of 1/50 (*w*/*w*), which was incubated at 37 °C for 18 h. The reaction was stopped by adding 1% of the final volume of TFA solution (trifluoroacetic acid) to halt trypsin activity [31]. 

The cleaning (clean up) and elution of the sample was carried out to bind proteins and remove impurities from the sample before mass spectrum analysis. Elution was carried out by adding 20 μL of elution buffer (70% acetonitrile). The elution and clean up processes were carried out using a C18 Spin Column (Thermo Scientific, Pierce Biotechnology, N Meridian Rd, Rockford, IL, USA), which each contained a porous C18 reserved phase resin that could effectively bind peptides.

### 2.5. Peptide Fractionation and Liquid Chromatography–Mass Spectrometry (LC-MS/MS) Analysis

**Figure 1:**
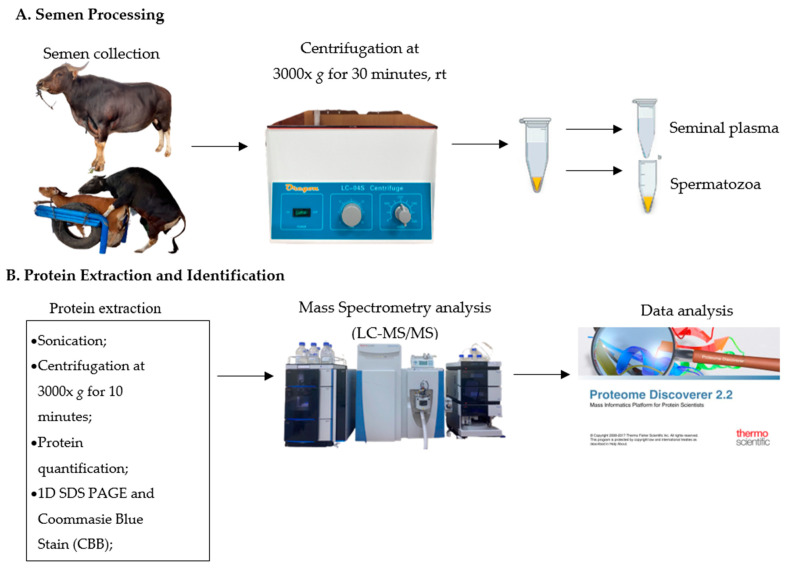
Scheme of the proteomic analysis of Bali bull seminal plasma. (**A**) Semen collection process for Bali bulls. (**B**) Protein extraction and identification.

Peptides from each sample were analyzed using LC-MS/MS on an Ultimate 3000 Nano LC system coupled to a Q-Exactive Plus Orbitrap HRMS (Thermo Fisher Scientific, Bremen, Germany) system with a PepMap-RSLC C18 column (75 m inner diameter, 15 cm, 3 µm, 100 pore size), part number ES 800 (Thermo Fisher Scientific, Bremen, Germany), at a flow rate of 300 nL/min. The elution process of peptides was carried out on the column with the following procedure: 0–3 min gradient of solvent B (0.1% formic acid, 98% acetonitrile); 2–35% solvent B for 3–30 min; 35–90% solvent B for 30–45 min; 90% solvent B for 45–90 min; and 5% solvent B for 60–90 min. Peptide signals were obtained using an LTQ-Orbitrap mass spectrometer (Thermo Fisher Scientific, Bremen, Germany). Survey scans in the 200–2000 *m*/*z* range were obtained with an MS resolution of 30,000 (at *m*/*z* 400) in the Orbitrap analyzer, followed by 10 intensive precursor MS/MS scans via collision-induced dissociation (CID) fragmentation at a normalized collision energy of 35% [21]. The LC-MS/MS workflow for the proteomic analyses of seminal plasma is shown in Figure 1.

### 2.6. Protein Identification

All of the data were obtained and processed using the Uniprot bovine protein database (http://www.uniprot.org) with the Proteome Discoverer version 2.2 software (Thermo Fisher Scientific). The identified proteins should contain at least one unique peptide per protein. The Gene Ontology (GO) annotation was then used to determine the functions and biological processes of the proteins in the dataset. The determination of pathways involved in seminal plasma was performed using the Panther (http://pantherdb.org accessed on 6 April 2022) and David databases (https://david.ncifcrf.gov/ accessed on 6 April 2022). Meanwhile, the interactions between proteins were retrieved from STRING (http://string-db.org accessed on 6 April 2022) [32]. The theoretical molecular weight of a protein was calculated based on the amino acid sequence in the database (proteins must have a sequence score HT > 0 and unique peptide ≥ 2).

## 3. Results

All of the proteins listed in the database were of *Bos taurus* origin due to the limited number of proteins from *Bos javanicus* in the database.

The proteins identified in the seminal plasma of Bali bulls were then analyzed using the STRING software. The color of the nodes indicates the interactions between proteins; light blue represents interactions derived from a curated database, and pink indicates experimentally defined interactions. The predicted interactions are represented by different colors; green for neighborhood genes implying functional linkage; red for fusion genes fused in some genomes (which are highly likely to be functionally linked); and dark blue for co-occurring genes that have a similar function. Other associations between proteins were identified in light green for text mining, black for co-expression to predict associations between genes, and blue for protein homology.

**Figure 2:**
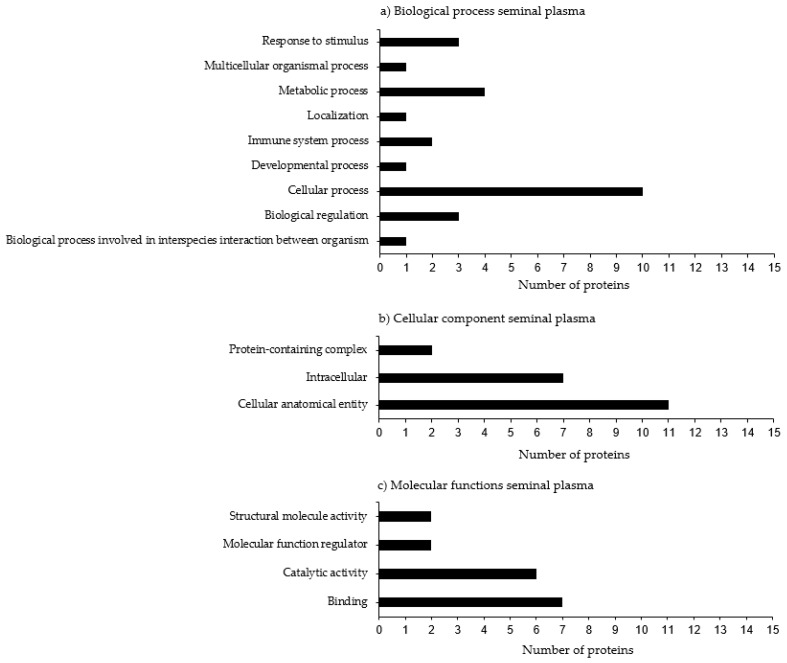
Gene Ontology terms of Bali bull seminal plasma protein based on (**a**) biological process, (**b**) cellular component, and (**c**) molecular function seminal plasma.

**Figure 3:**
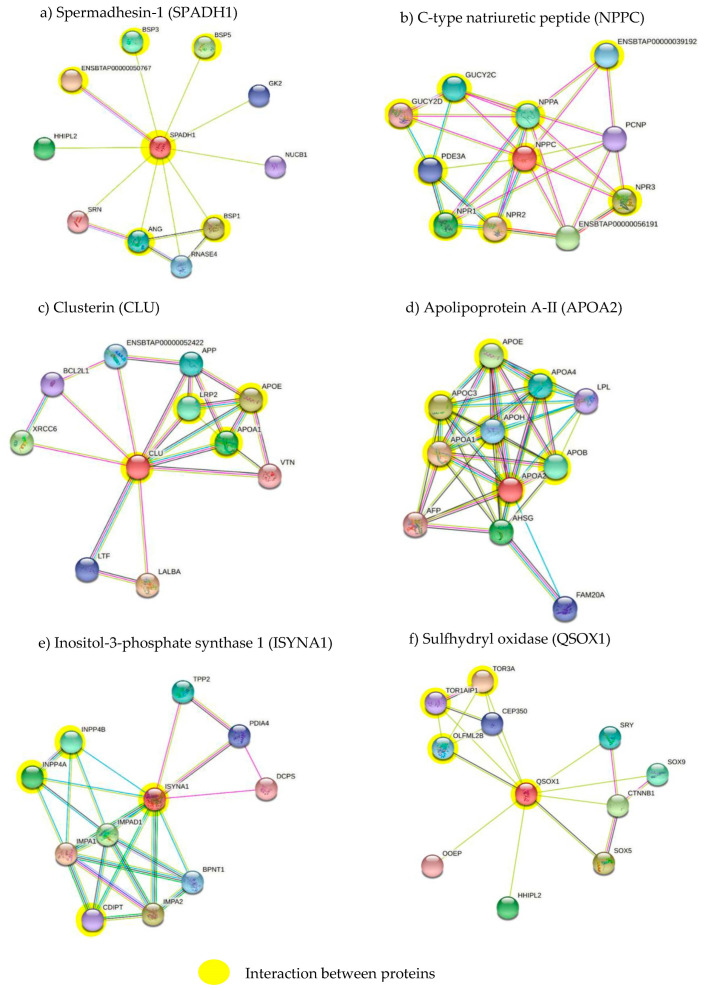
Interactions of the seminal plasma proteins related to sperm fertility: (**a**) Spermadhesin-1 (SPADH1); (**b**) C-type natriuretic peptide (NPPC); (**c**) clusterin (CLU); (**d**) apolipoprotein A-II (APOA2); (**e**) inositol-3-phosphate synthase 1 (ISYNA1); (**f**) sulfhydryl oxidase (QSOX1).

A total of 83 proteins had already been characterized by UniProt, and 11 proteins were uncharacterized. Protein functional annotations from Bali bulls were analyzed using PANTHER software (Figure 2). The most important biological processes associated with proteins identified in the seminal plasma were: the cellular process (10%), followed by metabolic processes (4%), response to stimulus and biological regulation (3%), and multicellular organismal processes, localization, and biological processes involved in interspecies interactions between organisms (1%). The cellular components of seminal plasma were mainly reported as cellular anatomical entity (11%), intracellular (7%), and protein-containing complex (2%). The molecular functions of the seminal plasma proteins were binding (7%), catalytic activity (6%), structural molecule activity, and regular molecular functions (2%). The protein STRING analysis revealed that SPADH1 interacted with several bovine seminal plasma proteins, NUCB1, GK2, HHIPL2, SRN, ANG, and RNASE4 (Figure 3a). The C-type natriuretic peptide protein interacted with its receptors and with the PEST proteolytic signal-containing nuclear protein (Figure 3b). The protein clusterin showed linkages with apolipoprotein A-I, LTF, LALBA, XRCC6, BCL2L1, APP, APOE, and VTN (Figure 3c). APOH interacted with several glycoproteins (APOE, APOA4, APOC3, APOA1, APOA2, APOB, AHSG), alpha-fetoprotein, and lipoprotein lipase (Figure 3d). ISYNA1 interacted with TPP2, PDIA4, DCPS, BPNT1, IMPA1, IMPA2, and IMPAD1 (Figure 3e). Finally, QSOX1 interacted with transcription factors OLFML2B, and TOR1AIP1 (Figure 3f).

## 4. Discussion

A total of 94 proteins were identified in the seminal plasma of Bali bulls analyzed using LC-MS/MS (Table S1). The total proteins in this study indicate that the distribution of proteins based on molecular weight in Bali bulls ranged from <11 to 110 kDa. Size exclusion chromatography relies on calibration with known molecular weight standards, determined using independent approaches, to provide information on the molecular weight distributions. A broad variety of biological processes has been described for seminal plasma proteins (i.e., cellular process, metabolic process, biological regulation, response to stimulus, immune system process, biological process involved in interspecies interactions between organisms, developmental process, localization, and the processes of multicellular organisms). Meanwhile, the cellular components include protein-containing complexes, intracellular and cellular anatomical entities, and the molecular functions consisted of binding, catalytic activity, molecular function regulation, and structural molecule activity. The proteins involved in regulating molecular functions in this study were proteins containing the TIMP2 and SERPIN domains. These functions of seminal plasma proteins are obviously related to their functions in sperm cells such as sperm motility, fertilization, and sperm capacitation.

**Figure 4:**
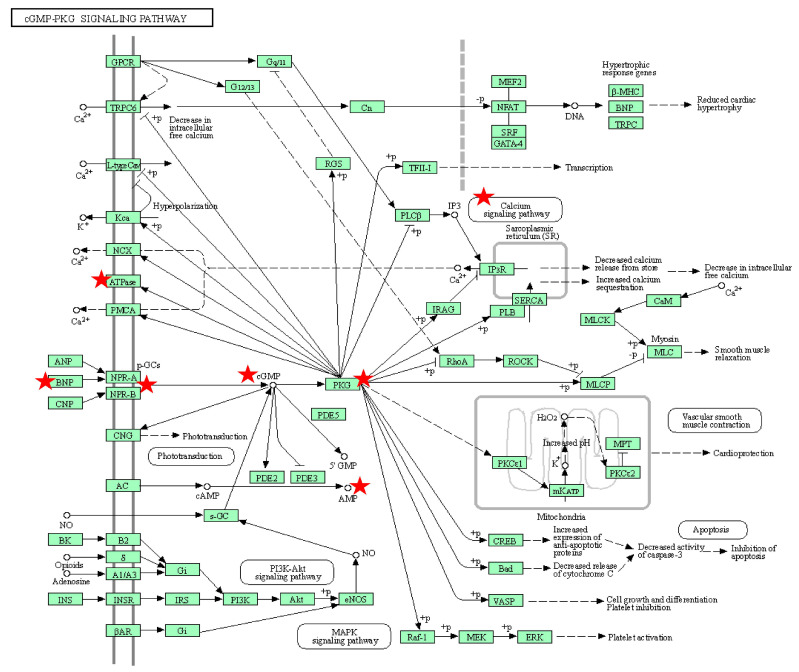
The cGMP-PKG signaling pathway. The activity of CGMP-PKC is activated through the binding of the CNP ligand (2’,3’-cyclic-nucleotide 3’-phospodiesterase) with the NPR-B receptor (guanylate cyclase). The response of the activation mechanism causes the conversion of adenosine triphosphate (ATP) into cAMP, which is important for the movement of spermatozoa.

The results of the analysis show that the seminal plasma protein expression pattern of Bali bulls is consistent with a functional reproductive capacity (Figure 3). Proteins such as spermadhesin 1 (SPADH1), C-type natriuretic peptide (NPPC), clusterin (CLU), apoliprotein A-II (APOA2), inositol-3-phosphate synthase 1 (ISYNA1), and sulfhydryl oxidase 1 (QSOX1) play roles in the reproductive functions of spermatozoa such as spermatozoa motility, capacitation, and acrosome reactions. The SPADH1 protein interacts with sperm protein binders (BSPs) such as BSP1, BSP3, and BSP5, which can act to influence spermatozoa motility through the cGMP-PKC signaling pathway (Figure 3). The results of analyses using DAVID (Figure 4) showed the activity of PKC, which was activated through the binding of the CNP ligand (2’,3’-cyclic-nucleotide 3’-phospodiesterase) with the NPR-B receptor (guanylate cyclase). The response of the activation mechanism caused the conversion of adenosine triphosphate (ATP) into cAMP, which is important for the movement of spermatozoa. The cAMP/protein kinase-A pathway is involved in the regulation of sperm motility [33]. BSP1 is a molecule that helps in the process of wrapping, transporting, and protein assembly [34], and is associated with spermatozoa motility [19]. BSPs are also involved in plasma membrane modification during sperm capacitation [3]. After the ejaculation process, BSPs can interact with choline phospholipids in the plasma membrane of spermatozoa and increase capacitation [35]. The BSP1 protein in bovine seminal plasma, expressed at high concentrations, plays a part in increasing spermatozoa motility through activation or an increased Ca^2+^-ATPase activity [36]. The interaction between BSPs and heparin allows for the acrosomal reaction to occur by activating a signaling cascade that can increase tyrosine phosphorylation [37]. BSP1 can also act as an inhibitor in the activation of protein kinase C (PKC), which is involved in the signaling leading to the induction of acrosomal reactions [38]. BSP5 belongs to the BSP family, together with BSP1 and BSP3, representing approximately 60% of all seminal plasma proteins in bulls [39,40,41,42]. 

NPPC protein expression in seminal plasma from Bali bulls plays an important role in influencing spermatozoa motility and initiating intracellular cGMP in cattle through binding to its receptor (NRP-B) [19]. The results obtained from the STRING analysis revealed that NPPC interacts with several proteins (Figure 3b), which can affect the molecular function (ATP binding) and biological response (cGMP biosynthetic process and regulation of oocyte development). All of these functions play an important part in the fertility of Bali bull spermatozoa. The role of the NPPC protein in influencing the motility of Bali bull spermatozoa through cGMP activation is related to the ATPase enzyme, which is important for spermatozoa movement (Figure 3b).

The results show that the CLU protein interacted with the APOA1 protein, which could mediate the motility of spermatozoa through the SPAP complex, and this was associated with the supply of low-density lipoprotein (LDL) (Figure 3c). The expression of the CLU protein in this study was different from that reported by [19], who stated that the CLU protein was found in the seminal plasma of bulls with low fertility. This difference is possibly because CLU can interact with other proteins with different functions and processes including decreasing the fertility of spermatozoa. CLU is produced in the testes, epididymis, and seminal vesicles, and has been used as a marker of oxidative stress for seminal plasma in humans [43]. Furthermore, these authors also reported that the increased levels of CLU in semen were positively correlated with DNA damage. The APOA1 protein is part of the high-density lipoprotein (HDL) complex that can interact with proteins located on the flagella and acrosomes in spermatozoa [44].

The results of the STRING analysis showed that APOA2 is a precursor protein that plays a role in the function of spermatozoa’s capacity for binding with the zona pellucida. APOA2 interacts with the proteins APOA1, APOE, APOH, APOB, APOC3, LPL, and APOA4, which are involved in plasma membrane modulation and capacitation (Figure 3d). The APOA1 protein is part of the high-density lipoprotein (HDL) complex that can interact with proteins located on the flagella and acrosomes in spermatozoa [44]. The subsequent catabolism of APOA2, determining the plasma HDL levels and their presence, is an important signal for specific interactions with HDL receptors such as cubilin or heat shock protein 60 (Hsp-60) [45]. Hsp-60 affects spermatozoa during cell maturation, and is detected in the center of the spermatozoa [45]. APOA2 was also identified as a multi-functional protein associated with oxidative stress response and sperm DNA fragmentation (SDF) [46].

Here, the ISYNA1 protein is the first to be reported as expressed in Bali bull seminal plasma, which could be related to the process of phospholipid biosynthesis. The ISYNA1 protein can interact with INPP4A, INPP4B, and CDIPT (Figure 3e) to convert glucose 6-phosphate to myoinositol 1 phosphate (I1P). ISYNA1 also catalyzes the synthesis of D-inositol-3-phosphate from glucose-6-phosphate (G6P) [47]. ISYNA1 is expressed in the testes, especially on Sertoli cells, which may be associated with spermatozoa fertility [48]. Sertoli cells can sense a change in osmolarity, and then induce ISYNA1 to help create that change in osmolarity. ISYNA1 inhibition may result in decreased spermatogenesis. The regulation of ISYNA1 expression was performed by p53 [49]; p53 was subsequently found to have a role in regulating spermatogenesis [50].

The QSOX1 protein interacts with TOR3A, OOEP, and HHPL2 (Figure 3f). QSOX1 contributes to the reduction in molecular oxygen into hydrogen peroxide, and then forms sulfide bonds in proteins and peptides [51]. QSOX1 also plays a part in the sperm’s response to oxidative stress [19]. Meanwhile, in the male reproductive tract, QSOX1 is involved in maintaining the structure and function of sperm through sulfhydryl oxidation, which can damage cells [52]. Furthermore, QSOX plays an important role in sperm physiological processes, and its regulation is associated with defects that may occur during spermatogenesis in hamsters [53] and mice [54].

Cyclic GMP (cGMP) is an intracellular agent that mediates nitric oxide (NO) and natriuretic peptides (NPs), and it regulates a wide variety of biological processes (Figure 4). Increased intracellular cGMP levels provide physiological signals through two cycle pathways, namely, the cGMP-protein kinase G (PKG), cGMP-regulated phosphodiesterase (PDE2, PDE3), and cGMP-gated cation channels, among which PKG is the main mediator. The specific activation of PKG1 from the formed substrate leads to a reduction in the cytosolic calcium concentration and a decrease in myofilament sensitivity to Ca^2+^ (Ca^2+^ desensitization). Qu et al. [55] found that the zinc-alpha-2-glycoprotein in sperm can bind to spermatozoa and speed up their movement through the cAMP pathway.

There are several important limitations to this study. First, this study used Bali bulls selected by the Center for Artificial Insemination for their good fertility, so we did not compare these with infertile bulls. Second, due to limited tools, we did not use 2D-SDS-PAGE, but 1D-SDS-PAGE can also provide accurate results that confirm the molecular weights of the proteins expressed in seminal plasma.

We realize that the research process should minimize data bias by anticipating the obstacles that will be encountered. However, our study was the first to report on Bali bulls, thereby providing great opportunities for further research.

## 5. Conclusions

In conclusion, this study provides new information about the protein content of seminal plasma in Bali bulls. The LC-MS/MS-based proteome approach that we applied in this study obtained 94 proteins. Based on analysis, the proteins derived from seminal plasma that are related to reproductive function are SPADH1, NPPC, CLU, APOA2, ISYNA1, and QSOX1. Studies of the seminal plasma proteins of Bali bulls can provide an understanding of the mechanisms that regulate male fertility, which in turn can have an impact on the success of artificial insemination. 

## Data Availability

Data supporting the reported results are contained within the article. All of the datasets collected and analyzed during the current study are available from the corresponding author on reasonable request.

## Supplementary Materials

The following supporting information can be downloaded at: https://www.mdpi.com/2076-2615/13/3/514/s1, Table S1: List of seminal plasma proteins in Bali bulls.
