# Supplementary material for: Protein Identification of Seminal Plasma in Bali Bull (Bos javanicus)
Source: Animals (Basel). 2023 Feb 1;13(3):514. doi: 10.3390/ani13030514 (PMC9913395; doi:10.3390/ani13030514)
Supplement: Supplementary file 1 [file animals-13-00514-s001.zip › animals-2133958-supplementary.pdf]

**Table S1.** List of seminal plasma proteins in Bali bulls.

| Accession number | Gene symbol | Description | Unique peptides | MW (kDa) | Molecular function |
|---|---|---|---|---|---|
| A0A4W2C0P5 | PAFAH 2 | Platelet-activating factor acetylhydrolase | 8 | 50.1 | platelet-activating factor acetyl transferase activity |
| A0A4W2C5E4 | N/A | CUB domain-containing protein | 1 | 15.3 | involved in fertilization |
| A0A4W2D8T3 | ALB | Albumin | 1 | 74 | DNA binding |
| A0A4W2GW93 | N/A | Uncharacterized protein | 1 | 73.9 | - |
| A0A3Q1LTS9 | LOC522 479 | SERPIN domain-containing protein | 2 | 42.7 | serine-type endopeptidase inhibitor activity |
| A0A3Q1MCG3 | NT5E | 5'-nucleotidase | 3 | 63 | catalytic activity |
| F6RP72 | LOC100 295712 | Tubulin alpha chain | 2 | 50.9 | major constituent of microtubules |
| A0A4W2BW23 | IGHM | Immunoglobulin heavy constant mu | 2 | 42.4 | immunoglobulin receptor binding |
| A0A3Q1N9Y5 | QSOX1 | Sulfhydryl oxidase | 4 | 86.1 | catalyzes the oxidation of sulfhydryl groups in peptide and protein thiols to disulfides |
| A0A4W2CDD2 | GPI | Glucose-6-phosphate isomerase | 2 | 63.6 | cytokine activity |
| A0A4W2C529 | CCT6A | Chaperonin containing TCP1 subunit 6A | 2 | 57.9 | ATP binding |
| A0A4W2C7S4 | HSPD1 | 60 kDa heat shock protein, mitochondrial | 1 | 61.1 | ATP hydrolysis activity |
| G5E531 | TCP1 | T-complex protein 1 subunit alpha | 1 | 60.2 | binding of sperm to zona pellucida |
| A0A4W2DQN7 | N/A | Uncharacterized protein | 1 | 44.7 | - |
| A0A4W2ELA6 | CPQ | Carboxypeptidase Q | 1 | 53 | carboxypeptidase that may play a pivotal role in the hydrolysis of circulating peptides |
| A0A4W2EBV6 | LOC113 905846 | Placenta-expressed transcript 1 protein | 1 | 30.2 | cell differentiation |
| | | | | | |
| A0A4W2H1Z5 | CCT8 | Chaperonin containing TCP1 subunit 8 | 1 | 60.2 | unfolded protein binding |
| A0A3Q1MGZ4 | TKFC | Triokinase/FMN cyclase | 1 | 64.1 | acetol kinase activity |
| A0A4W2EN64 | BPI | Bactericidal/permeability-increasing protein | 1 | 57.8 | lipopolysaccharide binding |
| A0A4W2DG58 | ISYNA1 | Inositol-3-phosphate synthase 1 | 1 | 70.7 | glucocycloaldolase activity |
| Q28068 | CCIN | Calicin OS=*Bos taurus* | 1 | 66.8 | morphogenic cytoskeletal element in spermiogenic differentiation |
| Q0P569 | NUCB1 | Nucleobindin-1 | 1 | 54.9 | calcium homeostasis |
| Continued | | | | | |

| Accession number | Gene symbol | Description | Unique peptides | MW (kDa) | Molecular function |
|---|---|---|---|---|---|
| A0A4W2G410 | CES5A | Liver carboxylesterase-like | 1 | 107.4 | hydrolase activity |
| A0A4W2CEF3 | GAPDHS | Glyceraldehyde-3-phosphate dehydrogenase | 1 | 44.1 | NADP binding |
| Q3T0K2 | CCT3 | T-complex protein 1 subunit gamma | 1 | 60.5 | assists the folding of proteins upon ATP hydrolysis |
| F1MU43 | ODF2 | Outer dense fiber protein 2 | 1 | 100.6 | major component of outer dense fiber of the sperm tail |
| G3MZ71 | KRT2 | Keratin 2 | 1 | 64.4 | cytoskeletal protein binding |
| A0A4W2HYV1 | HEXB | Beta-hexosaminidase | 3 | 61.2 | catalytic activity |
| A0A4W2D7P5 | AHSG | Alpha 2-HS glycoprotein | 4 | 39.7 | cysteine-type endopeptidase inhibitor activity |
| A0A4W2C5E4 | N/A | CUB domain-containing protein | 2 | 15.3 | involved in fertilization |
| A0A4W2I5U3 | APOA2 | Apolipoprotein A2 | 1 | 11.2 | receptor-binding of apolipoprotein |
| P17697 | CLU | Clusterin | 4 | 51.1 | extracellular chaperone that prevents aggregation of non-native proteins |
| A0A4W2I285 | N/A | CUB domain-containing protein | 1 | 16.2 | involved in fertilization |
| A0A4W2DYT6 | SERPINA1 | SERPIN domain-containing protein | 3 | 46.1 | serine-type endopeptidase inhibitor activity |
| A0A4W2GW93 | N/A | Uncharacterized protein | 4 | 73.9 | - |
| A0A4W2GQW1 | N/A | GLOBIN domain-containing protein | 1 | 16.7 | transporting oxygen |
| A0A4W2DL43 | PTGR1 | Prostaglandin reductase 1 | 1 | 46.1 | plays a role in the metabolic detoxification of alkenals and ketones |
| E1BNS9 | LDHC | L-lactate dehydrogenase | 1 | 36 | flagellated sperm motility |
| A0A4W2C2A5 | DNASE2L3 | Deoxyribonuclease | 1 | 35.2 | DNA binding |
| Q32LG3 | MDH2 | Malate dehydrogenase, mitochondrial | 1 | 35.6 | homodimerization activity |
| A0A4W2IIR6 | GC | GC vitamin D binding protein | 1 | 59.5 | involved in vitamin D transport and storage |
| A0A4W2IKM7 | N/A | Uncharacterized protein | 1 | 44.8 | - |
| A0A4W2D7P5 | AHSG | Alpha 2-HS glycoprotein | 3 | 39.7 | cysteine-type endopeptidase inhibitor activity |
| F1N430 | TIMP2 | Metalloproteinase inhibitor 2 | 2 | 26.1 | metalloendopeptidase inhibitor activity |
| A0A4W2CIX0 | N/A | Uncharacterized protein | 2 | 21.3 | - |

Continued

| Accession number | Gene symbol | Description | Unique peptides | MW (kDa) | Molecular function |
|---|---|---|---|---|---|
| A0A4W2C5E4 | N/A | CUB domain-containing protein | 1 | 15.3 | involved in fertilization |
| F1MNH9 | N/A | Uncharacterized protein | 1 | 12 | - |
| A0A4W2EL41 | PARK | Parkinsonism associated deglycase | 1 | 20.2 | as a positive regulator of androgen signaling pathway as well as cell growth and transformation |
| A0A4W2C3L7 | GPX6 | Glutathione peroxidase | 2 | 25 | glutathione peroxidase activity |
| P37141 | GPX3 | Glutathione peroxidase 3 | 2 | 25.6 | protects cells and enzymes from oxidative damage |
| A0A3Q1LI44 | N/A | Ig-like domain-containing protein | 1 | 15.1 | immune response |
| A0A4W2I285 | N/A | CUB domain-containing protein | 1 | 16.2 | involved in fertilization |
| A0A4W2BT41 | LOC101 908350 | Lipocln_cytosolic_FA-bd_dom domain-containing protein | 1 | 19.6 | odorant binding |
| G3MX65 | WFDC2 | WAP four-disulfide core domain 2 | 1 | 17.6 | peptidase inhibitor activity |
| A0A4W2HSN3 | LCN2 | Lipocalin 2 | 1 | 22.9 | enterobactin binding |
| A0A4W2I9W7 | RAB2A | RAB2A, member RAS oncogene family | 1 | 23.5 | GDP binding |
| A0A4W2DWN4 | N/A | Glutathione peroxidase | 1 | 25.1 | response to oxidative stress |
| A0A4W2C2A5 | DNASE 1L3 | Deoxyribonuclease | 1 | 35.2 | DNA binding |
| A0A4W2DX84 | C1qDC | C1q domain-containing protein | 1 | 39.5 | recognition receptor in innate immunity |
| A0A3Q1N9Y5 | QSOX1 | Sulfhydryl oxidase | 1 | 86.1 | reduction of oxygen to hydrogen peroxide |
| P29392 | SPADH 1 | Spermadhesin-1 | 4 | 15 | stimulates cell division and progesterone secretion of bovine granulosa cells in vitro in a potent and dose-dependent manner |
| A0A4W2C5E4 | N/A | CUB domain-containing protein | 2 | 15.3 | involved in fertilization |
| A0A4W2CIX0 | N/A | Uncharacterized protein | 2 | 21.3 | - |
| A0A4W2D7P5 | AHSG | Alpha 2-HS glycoprotein | 2 | 39.7 | cysteine-type endopeptidase inhibitor activity |
| A0A452DJ99 | NPPC | C-type natriuretic peptide | 1 | 20.3 | hormone activity |
| A0A4W2BT41 | LOC101 908350 | Lipocln_cytosolic_FA-bd_dom domain-containing protein | 1 | 19.6 | odorant binding |
| Continued | | | | | |

| Accession number | Gene symbol | Description | Unique peptides | MW (kDa) | Molecular function |
|---|---|---|---|---|---|
| A0A4W2C0P5 | PLA2G7 | Platelet-activating factor acetylhydrolase | 5 | 50.1 | platelet-activating factor acetyltransferase activity |
| A0A4W2CFP1 | N/A | Uncharacterized protein OS=Bos indicus x Bos taurus OX=30522 PE=4 SV=1 | 1 | 17.3 | - |
| A0A4W2CFU9 | N/A | Uncharacterized protein | 1 | 17.7 | - |
| A0A4W2C703 | LOC113879927 | SERPIN domain-containing protein | 6 | 49.2 | serine-type endopeptidase inhibitor activity |
| A0A3Q1LTS9 | LOC522479 | SERPIN domain-containing protein | 4 | 42.7 | serine-type endopeptidase inhibitor activity |
| A0A4W2D8T3 | ALB | Albumin | 2 | 74 | DNA binding |
| A0A4W2C5E4 | N/A | CUB domain-containing protein | 1 | 15.3 | involved in fertilization |
| A0A4W2I285 | N/A | CUB domain-containing protein | 1 | 16.2 | involved in fertilization |
| A0A4W2GW93 | N/A | Uncharacterized protein | 1 | 73.9 | - |
| A0A4W2DQN7 | N/A | Uncharacterized protein | 2 | 44.7 | - |
| A0A4W2CDD2 | GPI | Glucose-6-phosphate isomerase | 3 | 63.6 | regulation of endothelial cell migration |
| A0A3Q1N9Y5 | QS0X1 | Sulfhydryl oxidase | 5 | 86.1 | the reduction of oxygen to hydrogen peroxide |
| A0A4W2CZ37 | BPIFB1 | BPI fold-containing family B member 1 | 2 | 52.3 | lipid binding |
| A0A4W2D7P5 | AHSG | Alpha 2-HS glycoprotein | 1 | 39.7 | cysteine-type endopeptidase inhibitor activity |
| A0A4W2CCZ7 | LOC113879069 | Beta-hexosaminidase | 2 | 62 | beta-N-acetylhexosaminidase activity |
| A0A4W2ELA6 | CPQ | Carboxypeptidase Q | 1 | 53 | plays an important role in the hydrolysis of circulating peptides |
| A0A4W2DMT7 | ST6GAL1 | ST6 beta-galactoside alpha-2,6-sialyltransferase 1 | 1 | 46.2 | protein homodimerization activity |
| A0A4W2EBV6 | LOC113905846 | Placenta-expressed transcript 1 protein | 1 | 30.2 | cell differentiation |
| A0A4W2G410 | CES5A | Liver carboxylesterase-like | 2 | 107.4 | hydrolase activity |
| F6RP72 | N/A | Tubulin alpha chain | 1 | 50.9 | major constituent of microtubules |
| A0A3Q1MGZ4 | TKFC | Triokinase/FMN cyclase | 1 | 64.1 | represses IFIH1-mediated cellular antiviral response |
| A0A4W2G4G5 | CTSA | Carboxypeptidase | 1 | 55.8 | regulation of protein stability |
| A0A3Q1MCG3 | NT5E | 5'-nucleotidase | 3 | 63 | catalytic activity |
| Continued | | | | | |

| Accession number | Gene symbol | Description | Unique peptides | MW (kDa) | Molecular function |
|---|---|---|---|---|---|
| A0A4W2E5S2 | SIAE | Sialic acid acetylesterase | 1 | 72.1 | regulation of immune system process |
| A0A4W2F121 | HSPA13 | Heat shock protein family A (Hsp70) member 13 | 1 | 52.8 | ATP binding |
| A0A4W2EN64 | BPI | Bactericidal permeability increasing protein | 1 | 57.8 | immune response |
| Q3ZBH0 | CCT2 | T-complex protein 1 subunit beta | 1 | 57.4 | protein stabilization |

*Protein detected on LC-MS/MS; MW: molecular weight.
Source: https://www.uniprot.org/
